# Air pollutants and daily number of admissions to psychiatric emergency services: evidence for detrimental mental health effects of ozone

**DOI:** 10.1017/S2045796019000623

**Published:** 2019-11-06

**Authors:** F. Bernardini, L. Attademo, R. Trezzi, C. Gobbicchi, P.M. Balducci, V. Del Bello, G. Menculini, L. Pauselli, M. Piselli, T. Sciarma, P. Moretti, A. Tamantini, R. Quartesan, M.T. Compton, A. Tortorella

**Affiliations:** 1 Department of Mental Health, AAS5 ‘Friuli Occidentale’, Pordenone, Italy; 2 Department of Mental Health, ASP Basilicata, Potenza, Italy; 3 Research and Statistics Division, Board of Governors of the Federal Reserve System, Washington, DC, USA; 4 Department of Mental Health, AUSL Umbria 2, Terni, Italy; 5 Division of Psychiatry, Department of Medicine, University of Perugia, Perugia, Italy; 6 Columbia University, College of Physicians & Surgeons, New York, USA; 7 Functional Area of Psychiatry, University of Perugia, Perugia, Italy

**Keywords:** Air pollution, emergency service, environmental pollutants, hospital, mental health

## Abstract

**Aims:**

Aim of the current study is to investigate the associations between daily levels of air pollutants (particulate matter, ozone, carbon monoxide, nitrogen dioxide) and daily admissions for mental disorders to the emergency department of two general hospitals in Umbria region (Italy).

**Methods:**

We collected data about daily admissions to psychiatric emergency services of two general hospitals, air pollutants' levels and meteorological data for the time period 1 January 2015 until 31 December 2016. We assessed the impact of an increase in air pollutants on the number of daily admissions using a time-series econometric framework.

**Results:**

A total of 1860 emergency department admissions for mental disorders were identified. We observed a statistically significant impact of ozone levels on daily admissions. The estimated coefficient of O_3_ is statistically significant at the 1% level. All other pollutants were not significantly associated with the number of daily admissions.

**Conclusions:**

Short-term exposure to ozone may be associated with increased psychiatric emergency services admissions. Findings add to previous literature on existing evidence for air pollution to have an impact on mental health. Ozone may be considered a potential environmental risk factor for impaired mental health.

## Introduction

Air pollution is a major environmental risk to health, concerning both developed and developing countries, with diverse and substantial public health implications (WHO, 2018*a*). Ambient (outdoor) air pollution in both cities and rural areas was estimated to cause 4.2 million premature deaths worldwide in 2016. In that same year, 91% of the world's population was living in places where the WHO Air Quality Guidelines levels were not met.

The WHO Guidelines provide an assessment of health effects of air pollution and thresholds for health-harmful pollution levels. The Guidelines apply worldwide and are based on expert evaluation of current scientific evidence for particulate matter (PM), ozone (O_3_), nitrogen dioxide (NO_2_) and sulphur dioxide (SO_2_), in all WHO regions.

By decreasing air pollution levels, countries can reduce disease burden related to stroke, heart disease, lung cancer and both chronic and acute respiratory diseases, including asthma. Furthermore, it has been recently hypothesised that exposure to xenobiotic heavy metals such as lead and cadmium – constituents of air pollution such as particulate matter and nitrogen and sulphur oxides, organic solvents and other environmental pollutants – could be component causes for schizophrenia and other psychotic disorders (Attademo *et al*., 2017). Moreover, recent studies in Sweden found that ambient air particle concentrations are associated with the number of visits to psychiatric emergency units in the warm season, suggesting that air pollution may exacerbate an underlying psychiatric disorder, or increase mental distress, even in areas with comparatively low levels of air pollution (Oudin *et al*., 2018). Similarly, long-term exposure to ambient air pollution could be an independent risk factor for mental health disorders ranging from subjective stress to depressive disorders and suicidal ideation (Shin *et al*., 2018). Recent studies analysing the association between daily levels of air pollutants and hospital admissions for mental disorders showed significant results for different pollutants considered both for admissions for generic mental disorders (Chen *et al*., 2018; Kim *et al*., 2018; Song *et al*., 2018; Lee *et al*., 2019; Qiu *et al*., 2019) and for specific diagnoses such as schizophrenia (Gao *et al*., 2017; Duan *et al*., 2018; Bai *et al*., 2019), depression (Szyszkowicz *et al*., 2016; Wang *et al*., 2018) and substance abuse (Szyszkowicz *et al*., 2018).

In addition, climate change is a major threat to the health of our planet, and is closely linked to air pollution. Over the past 50 years, human activities such as the burning of fossil fuels have released sufficient quantities of carbon dioxide (CO_2_) and other greenhouse gases to affect the global climate. The resulting changes in the global climate bring a range of risks to health, from deaths in extreme high temperatures to changing patterns of infectious diseases (WHO, 2018*b*).

The health effects of climate change are vast and distressingly serious, involving almost all organ systems of humans (and most other biological fauna and flora). The mental health consequences are also vast, pervasive, and likely to last longer than most other impacts on health (Clayton *et al*., 2017).

The aim of the current study is to investigate the associations between daily levels of five air pollutants (particulate matter [PM_10_ and PM_2.5_], ozone [O_3_], carbon monoxide [CO], nitrogen dioxide [NO_2_]) and daily admissions for mental disorders to the emergency department of two general hospitals in Umbria region (Italy). Considering the daily number of admissions to psychiatric emergency services as a proxy measure of the psychiatric morbidity in the Umbria region, we hypothesised a positive association between air pollutant levels and daily number of psychiatric emergency admissions.

## Methods

### Psychiatric emergency services data

Data about daily admissions to psychiatric emergency services of Ospedale Santa Maria della Misericordia (Perugia) and Ospedale San Giovanni Battista (Foligno) were collected for the time period 1 January 2015 until 31 December 2016 (731 days). The catchment area covered by Ospedale Santa Maria della Misericordia (Perugia) consists of approximately 501 351 residents on an area of 4298.38 km^2^, with a population density of 116 inhabitants per km^2^ (ISTAT, 2015). The Ospedale San Giovanni Battista (Foligno) provides services for an area of about 901.77 km^2^, with 98 633 citizens and a population density of 109 inhabitants per km^2^ (ISTAT, 2015).

The psychiatric emergency services rely on the 24-h presence of a consultant psychiatrist in each of the above-mentioned hospitals. After the triage and first check in the Emergency Department, subjects presenting with an acute psychiatric condition undergo a screening visit operated by the mental health specialist. The number of consultations provided during each day of the considered time period was recorded into an electronic datasheet. Information about air pollution and meteorological data was then collected for each day and subsequently entered into the database.

### Air pollution data

Data about daily levels of respirable particular matter (PM_10_ and PM_2.5_), ozone (O_3_), carbon monoxide (CO) and nitrogen dioxide (NO_2_) were collected from the regional agency for environmental protection of Umbria (Arpa Umbria – Agenzia Regionale per la Protezione Ambientale dell'Umbria) datasets (ARPA, 2017). We used air monitoring data for the different types of air pollutants which were averaged across up to 16 monitor stations dispersed in the region. The maximum distance among monitor stations is 91 km.

### Meteorological data

Data about daily meteorological conditions such as daily average temperature, daily average pressure, daily average humidity and maximum and average wind speed in the study period were obtained from the meteorological station of Perugia-Monteluce, a centrally located urban station (monteluce.lineameteo, 2017).

### Data analyses

**Fig. 1.**
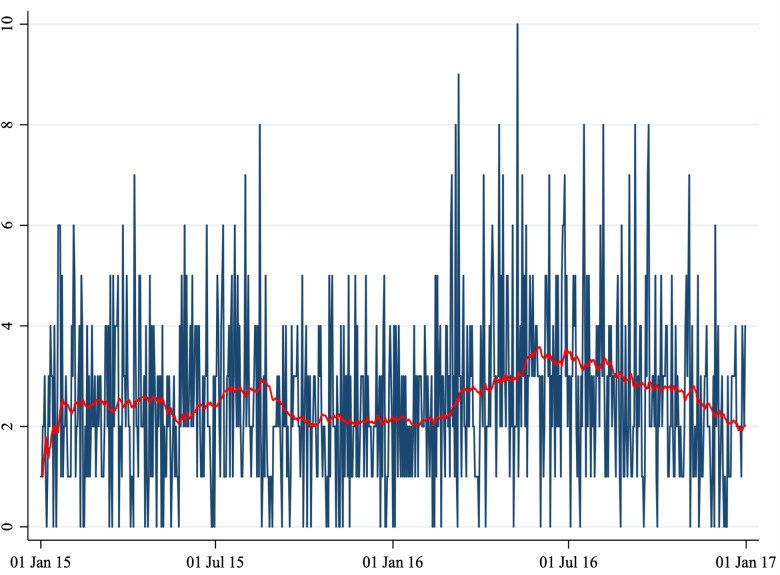
Number of daily admissions.

*Note*: This figure shows the number of daily admissions for psychiatric reasons to the Emergency Department of two hospitals (Perugia and Foligno) from 1 January 2015 to 31 December 2016. The red line shows a 20-periods (days) moving average.

**Fig. 2.**
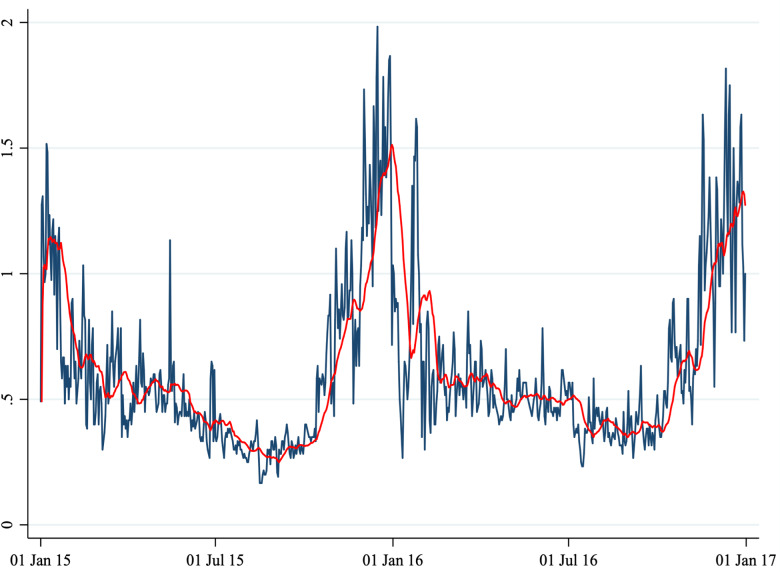
Average daily CO concentration.

*Note*: This figure shows the average daily CO concentration in Perugia and Foligno from 1 January 2015 to 31 December 2016. The red line shows a 20-period (days) moving average. The unit of measures of the *y*-axis is mg/m^3^.

**Fig. 3.**
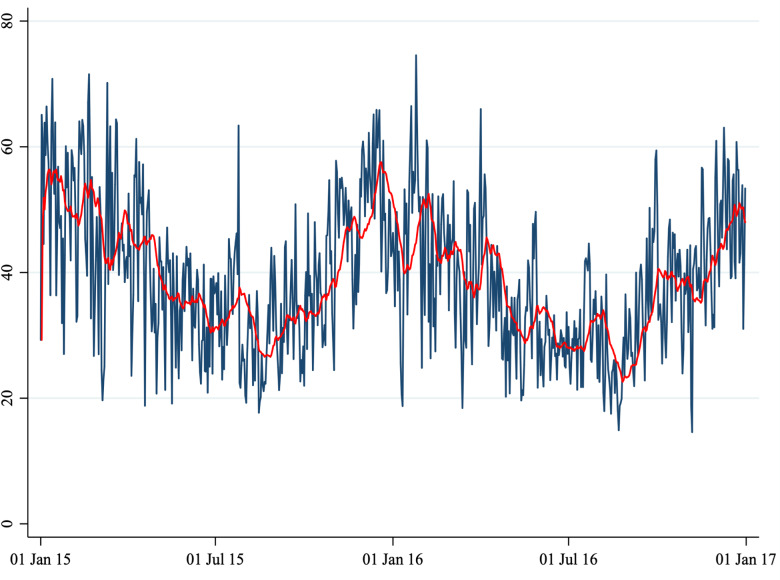
Average daily NO_2_ concentration.

*Note*: This figure shows the average daily NO_2_ concentration in Perugia and Foligno from 1 January 2015 to 31 December 2016. The red line shows a 20-period (days) moving average. The unit of measure of the *y*-axis is μg/m^3^.

**Fig. 4.**
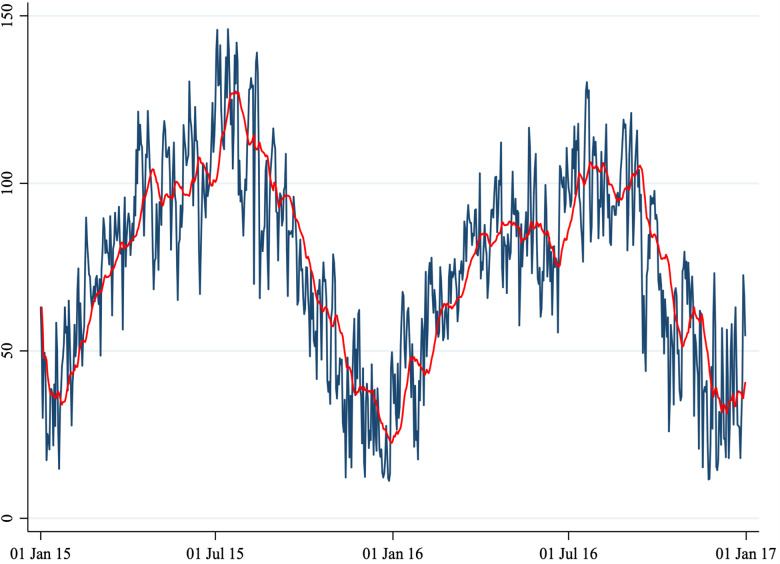
Average daily O_3_ concentration.

*Note*: This figure shows the average daily O_3_ concentration in Perugia and Foligno from 1 January 2015 to 31 December 2016. The red line shows a 20-period (days) moving average. The unit of measure of the *y*-axis is μg/m^3^.

**Fig. 5.**
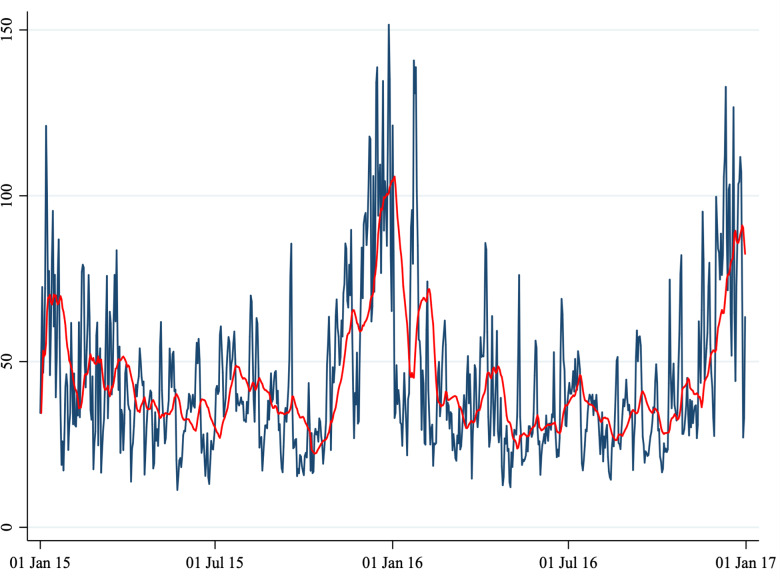
Average daily PM_10_ concentration.

*Note*: This figure shows the average daily PM_10_ concentration in Perugia and Foligno from 1 January 2015 to 31 December 2016. The red line shows a 20-period (days) moving average. The unit of measure of the *y*-axis is μg/m^3^.

**Fig. 6.**
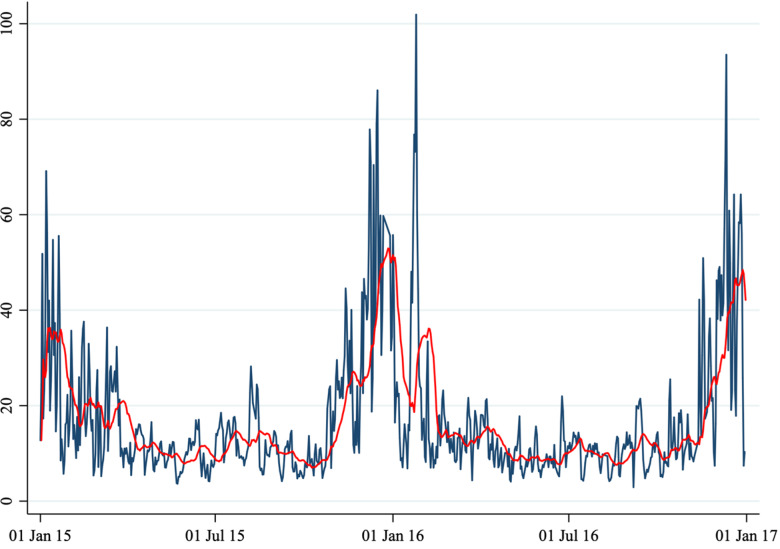
Average daily PM_2.5_ concentration.

*Note*: This figure shows the average daily PM_2.5_ concentration in Perugia and Foligno from 1 January 2015 to 31 December 2016. The red line shows a 20-period (days) moving average. The unit of measure of the *y*-axis is μg/m^3^.

We assessed the impact of an increase in air pollutants on the number of daily admissions using a time-series econometric framework. Specifically, we regressed the daily number of admissions (dependent variable) on the daily measure of air pollution, controlling for a set of covariates. Formally, the model can be expressed as:1
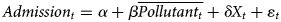
where *Admission*_*t*_ is the daily number of admissions, *α* is a constant term (intercept), *β* and *δ* are parameters, 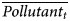 is a measure of daily air pollution, *X*_*t*_ is a matrix of controls and ɛ_*t*_ is a residual. As for the strictly exogenous variable 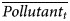, we considered five air pollutants: PM_10_, PM_2.5_, NO_2_, CO and O_3_ concentration levels. Because air pollutants show a seasonal pattern (Figs 1 to 6) we removed the seasonal mean (defined as a 20-period moving average) and considered the daily deviation from it. In other words, 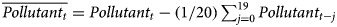. Our choice to define the seasonal mean as a 20-period moving average was discretionary but did not affect our results. Our evidence holds true with different choices such as allowing for seasonal dummy variables in Equation (1), considering a different length of the moving average, or considering the deviation from a biweight filter. The variable 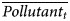 captures daily pollution concentration levels from their mean and it is strictly exogenous in Equation (1). The exogeneity of 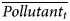 ensures that the estimated elasticity of daily admissions to air pollutants 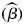 is unbiased. Finally, as for the matrix of controls 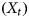 we included the following variables: (i) a set of dummy variables for the day of the week, (ii) a variable capturing the average daily temperature, (iii) a variable capturing the average daily pressure, (iv) a variable capturing the average daily wind speed and (v) a variable capturing average daily humidity. Also, we include the squares and cubes of (ii), (iii) and (iv) in order to control for possible non-linear effects of temperature, pressure, wind speed and humidity. All control variables appeared to be strictly exogenous in Equation (1).

Because of the (ordinal) nature of the dependent variable, we employed an ordered logit model (McCullagh, 1980; Greene, 2014) estimated using maximum likelihood. We ran the regressions including only lag 0 of the air pollutants. As robustness checks, we ran the same set of regressions using a linear regression model and using a Poisson regression model. Also, we ran a set of regressions allowing up to 10 lags of the air pollutant regressors. These robustness checks largely confirmed our baseline results.

In our econometric framework, we have ‘unified’ the two locations (emergency services of Ospedale Santa Maria della Misericordia in Perugia and Ospedale San Giovanni Battista in Foligno). Specifically, we have summed the total daily number of visits in both hospitals, and considered as pollutants the average daily entries across the two areas. As a robust check exercise we have also considered two alternative options: (i) using the average number of daily visits across the two locations as left-hand-side variable, and (ii) running a panel-data analysis (that is, considering the two locations as separate). In both cases, our results have been confirmed.

The statistical analysis was conducted using ‘STATA 13.0 – StataCorp LLC’.

## Results

A total of 1860 cases (1461 in Perugia and 399 in Foligno) of emergency department admissions for mental disorders were recorded over the study period. Summary statistics of the variables in our dataset are shown in Table 1. A correlation matrix is given in Table 2. Time series of the variables of interest are shown in Figs 1 to 6.

**Table 1.** Summary statistics

Variable	Unit of measure	No. of Obs.	Mean	s.d.	Min	Max
Admissions	Units	731	2.5	1.7	0	10
PM_10_	μg/m^3^	731	44.4	23.9	11.3	151.6
PM_2.5_	μg/m^3^	731	16.8	13.8	2.9	101.9
NO_2_	μg/m^3^	731	39.0	11.8	14.6	74.6
CO	mg/m^3^	731	0.62	0.33	0.17	1.98
O_3_	μg/m^3^	731	74.8	29.4	11.2	146
Temperature	°C	715	14.9	7.2	0.4	30.8
Pressure	hPa	715	1010.5	48.6	88.8	1031.6
Humidity	%	715	69.1	33.5	33.7	825
Wind	km/h	715	5.8	4.8	0	90

*Note*: This table shows descriptive statistics of the relevant variables in the dataset.

**Table 2.** Correlation matrix

	Admissions	PM_10_	PM_2.5_	NO_2_	CO	O_3_
Admissions	1.00					
PM_10_	−0.10	1.00				
PM_2.5_	−0.10	0.91	1.00			
NO_2_	−0.06	0.61	0.61	1.00		
CO	−0.12	0.81	0.83	0.62	1.00	
O_3_	0.14	−0.51	−0.58	−0.46	−0.73	1.00

*Note*: This table shows the Pearson's correlation coefficients among relevant variables in the dataset. *p*-values are as follows: for two pairs (admissions–PM_10_ and admissions–CO) are 0.01, for one pair (admissions–NO_2_) is 0.13, and for all others 0.00.

The number of daily admissions was negatively correlated with daily concentration levels of PM_10_, PM_2.5_, NO_2_ and CO, while they were positively correlated with O_3_ levels. Virtually all correlations were statistically significant at the 1% level of significance. The number of daily admissions to the Emergency Department for psychiatric reasons appears relatively stable over time (except for an increase in mid-2016), with daily peaks throughout the entire time series. On the other hand, air pollutants exhibit a clear seasonal pattern. Average daily CO concentration levels as well as NO_2_, PM_10_ and PM_2.5_ tend to be higher during the cold season, while O_3_ concentration levels tend to be higher during the warm season. For practical purposes, in Figs 1 to 6 we have identified the ‘seasonal mean’ with a 20-period (days) moving average (red line). For all air pollutants there is considerable daily variation around the seasonal mean, which should ensure enough variation for identification purposes.

Switching to econometric evidence, regression results are presented in Table 3. The first column in Table 3 reports the results of univariate (‘Uni’) regressions, that allow only one pollutant to enter into Equation (1). Overall, we observe a statistically significant impact of O_3_ levels on daily admissions. The estimated coefficient of O_3_ is statistically significant at the 1% level. According to our results, an increase of 1 µg/m^3^ of O_3_ concentration (relative to the average concentration of the last 20 days) results in 0.013 more admissions to the hospitals. While the estimated effect might seem small, it is in fact appreciable. Because the variable used in the regression (O_3_ concentration levels in deviation from a moving average) has a maximum of 52, we can deduce that in the days with a peak in O_3_ concentration levels, one admission (after rounding) at the hospitals can be explained by the increased pollution levels. Considering that the dependent variable (number of admissions) has a mean of 2.5 (1.7, standard deviation), the effect of increased O_3_ concentration levels do not seem trivial.

**Table 3.** Regressions results

	Uni	Biv	Biv	Biv	Biv	Biv	Biv	Biv	Biv	Biv	Biv	All
PM_10_	−0.005	−0.003	−0.004	0.001	−0.003							−0.001
	[0.004]	[0.007]	[0.004]	[0.005]	[0.004]							[0.007]
PM_2.5_	−0.009	−0.005				−0.007	0.001	−0.004				0.003
	[0.007]	[0.012]				[0.008]	[0.010]	[0.007]				[0.013]
NO_2_	−0.010		−0.005			−0.005			−0.003	−0.007		−0.004
	[0.009]		[0.010]			[0.011]			[0.010]	[0.009]		[0.011]
CO	−0.690			−0.725			−0.717		−0.649		−0.403	−0.435
	[0.345]*			[0.484]			[0.487]		[0.382]		[0.372]	[0.529]
O_3_	0.013				0.012			0.012		0.013	0.011	0.011
	[0.005]***				[0.005]***			[0.005]***		[0.005]***	[0.005]***	[0.005]***
Obs.	715	715	715	715	715	715	715	715	715	715	715	715

*Note*: This table shows the estimated coefficients of a set of regressions. Each column shows the results of a different regression. Standard errors are reported in brackets. ‘Uni’ stands for univariate model (for brevity, we show the results of all univariate regressions in a single column), that is a model in which we allow only one pollutant to enter into the regression. ‘Biv’ stands for bivariate, and ‘All’ stands for all variables. All regressions control for daily temperature, pressure, humidity, wind, their squares and cubes, and a set of dummies capturing the day of the week. Significance levels are as follows: *p < 0.1, **p < 0.05, ***p < 0.01.

All other pollutants are not significantly associated with the number of daily admissions. For CO we estimated an impact significant only at the 10% level but the sign of the coefficient is the opposite of the initial analysis (negative, instead of positive).

The following ten columns in Table 3 show the results of the bivariate (‘Biv’) regressions in which we allow two pollutants to enter into Equation (1). As for the ‘Uni’ regressions, this set of regressions allow for a set of control variables. Again, we observe significant coefficients for O_3_ levels at 1% level of significance. As for CO, in none of the regressions the estimated coefficient is significant, indicating that the result from the ‘Uni’ model is not robust. All other pollutants are estimated to have an effect not statistically different from zero. Finally, when we allow all pollutants (last column ‘All’ of Table 3) to enter into Equation (1), the coefficient of O_3_ remains stable and highly significant, and all other pollutants continue to remain statistically indistinguishable from zero.

As robustness checks, we repeated the analysis using (i) a linear regression model and (ii) a Poisson regression model. The evidence emerging from these alternative sets of regressions largely confirm the original findings. We have also run a set of regressions in which we have expanded the number of control variables and allowed a set of seasonal or monthly dummies to enter into Equation (1). In these regressions we obtained results virtually identical to the one presented in Table 3, as the seasonal/monthly dummies are estimated to be insignificantly different from zero. Finally, we ran a complementary set of regressions allowing up to 10 lags of each pollutant in Equation (1). The associations observed for lag 1 to lag 10 were generally lower than for lag 0 and were not statistically significant.

## Discussion

We observed a statistically significant association of daily ozone levels and daily number of admissions to psychiatric emergency services. Ozone is a component of photochemical smog and a powerful oxidant, and is considered as one of the most important air pollutants (Lauer, 2010). Environmental ozone exposure is associated with respiratory disorders such as loss of lung function, exacerbation of asthma and lung inflammation. Adverse effects within the central nervous system (CNS) have also been described (Martinez-Lazcano *et al*., 2013). A number of studies found that ozone may have a relevant interference with CNS physiology, that its exposure may be linked to brain disease and contribute to inflammation and oxidative stress (Block *et al*., 2012). Animal studies investigating the neurotoxic effects of ozone inhalation show that ozone exposure may reduce dopaminergic neurons, and increase lipid peroxidation (Pereyra-Munoz *et al*., 2006), vascular endothelial growth factor, interleukin-6, tumour necrosis factor *α* (Araneda *et al*., 2008) and c-Fos expression in different brain regions (Gackière *et al*., 2011). In rodents, short-term exposure results in cerebral oedema, neurodegeneration in the hippocampus, striatum and substantia nigra, and altered behaviour (Block *et al*., 2012); also, long-term exposure to low levels of ozone causes changes in microglial activation, changes in cell morphology in the substantia nigra and striatum, and loss of nigral dopaminergic neurons (Kirrane *et al*., 2015). In addition, it has been shown that ozone may interfere with cerebral blood vessels by modulating the expression of genes involved in brain vasoreactivity, affect the immune system, irritate mucous membranes and alter neurotransmitters concentrations such as serotonin (as well as dopamine). Other studies suggest that chronic ozone exposure causes cortical and hippocampal alterations that involve reduced oxygen suppression and catalase activity, and lowered central monoamine levels (Gładka *et al*., 2018).

In this line of thought, the abovementioned pathological alterations may be associated with a worsening in psychiatric disorders or mental distress, so that ozone may be considered a potential environmental risk factor for impaired mental health.

Furthermore, air pollutant levels may conceivably affect mental health conditions not only through biological or chemical mechanisms, but also through different mechanisms including psychological mechanisms (e.g. feeling anxious because of the elevated levels of air pollution often reported in the media).

Recent reviews of literature about epidemiological studies on ambient ozone exposure and mental health found evidence that ozone exposure may affect autism spectrum disorders, may lead to motor disorders (Parkinson's disease) and cognitive impairment (dementia), and may have an influence on the incidence of depression and suicide, although results of reviewed studies were inconclusive (Gładka *et al*., 2018; Zhao *et al*., 2018). In a 2016 study which used a methodology similar to our study (Szyszkowicz *et al*., 2016) ozone air levels showed an association with increased risk of an emergency department visit for depression in Canada. A 2007 study (Szyszkowicz, 2007) showed a significant increment in daily depression-related emergency department visits and ground level ozone for female patients in the warm season.

To the best of our knowledge, the current study is one of the first studies worldwide and the first study in Italy investigating the association between daily concentration of air pollutants and the daily number of visits to a psychiatric emergency unit.

Our results add to previous literature on existing evidence for air pollution to have a role in the cause or worsening of mental distress and psychiatric disorders (Szyszkowicz *et al*., 2009, 2010, 2016; Mehta *et al*., 2015; Power *et al*., 2015; Oudin *et al*., 2016, 2018; Attademo *et al*., 2017; Attademo and Bernardini, 2017; Bernardini *et al*., 2019).

Contrary to our hypothesis, we did not find a positive association between most of the air pollutants considered in our study, such as PM_10_, PM_2.5_, CO and NO_2_. This is in contrast with previous literature on this subject (Szyszkowicz *et al*., 2009, 2010, 2018; Mehta *et al*., 2015; Power *et al*., 2015; Chen *et al*., 2018; Duan *et al*., 2018; Eguchi *et al*., 2018; Kim *et al*., 2018; Oudin *et al*., 2018; Shin *et al*., 2018; Song *et al*., 2018; Bai *et al*., 2019; Lee *et al*., 2019; Qiu *et al*., 2019).

This could be explained by the methodological limitations of our study, which are several.

First, our sample size is smaller than most previous studies on air pollution exposure and mental disorders. Second, air pollution levels in Umbria region are lower than in China or big cities that were sources of most previous studies on this topic, resulting in limited comparability and generalisability of our findings. Third, we only report the number of emergency department admissions; we do not have data about clinical and socio-demographic characteristics of the admitted patients. Future studies should focus on elucidating the impact of air pollution levels in specific socio-demographic or diagnostic groups. Fourth, as in most previous time-series studies, we utilised measurements from fixed-site monitor stations as a proxy for the population pollutants exposure level, with the simplified assumption that each person has the same exposure. Exposure may vary depending on different factors such as location of home and workplace, occupational setting and time spent outdoors. Fifth, the study period was relatively short. Our study has also some notable strengths. First, we provide data concerning emergent (non-scheduled) admissions to emergency department of two hospitals, while some of the previous studies on this topic reported data about hospital admissions for mental disorders which included both emergent and scheduled admissions. Second, we used air monitoring data for five types of air pollutants which were averaged across 16 monitor stations dispersed in the region, which is more representative of the general population exposure than data from one single monitor station.

## Data

The data are available on request from the corresponding author.
